# INHIBITION OF TOLL-LIKE RECEPTOR 4 PROTECTS AGAINST INFLAMMATION-INDUCED MECHANOBIOLOGICAL ALTERATIONS TO INTERVERTEBRAL DISC CELLS

**DOI:** 10.22203/eCM.v041a37

**Published:** 2021-05-20

**Authors:** T.D. Jacobsen, P.A. Hernandez, N.O. Chahine

**Affiliations:** 1 Department of Biomedical Engineering, Columbia University, New York, NY; 2 Department of Orthopaedic Surgery, University of Texas Southwestern Medical Centre, Dallas, TX; 3 Department of Orthopaedic Surgery, Columbia University, New York, NY

**Keywords:** Intervertebral disc, mechanobiology, inflammation, cell mechanics, toll-like receptor-4, actin cytoskeleton, biophysical properties

## Abstract

Intervertebral disc (IVD) degeneration is associated with elevated levels of inflammatory cytokines implicated in disease aetiology and matrix degradation. Toll-like receptor-4 (TLR4) has been shown to participate in the inflammatory responses of the nucleus pulposus (NP) and its levels are upregulated in disc degeneration. Activation of TLR4 in NP cells leads to significant, persistent changes in cell biophysical properties, including hydraulic permeability and osmotically active water content, as well as alterations to the actin cytoskeleton. The study hypothesis was that inflammation-induced changes to cellular biomechanical properties and actin cytoskeleton of NP cells could be prevented by inhibiting TLR4 signalling. Isolated NP cells from bovine discs were treated with lipopolysaccharide (LPS), the best studied TLR4 agonist, with or without treatment with the TLR4 inhibitor TAK-242. Cellular volume regulation responses to step osmotic loading were measured and the transient volume-response was captured by time-lapse microscopy. Volume-responses were analysed using mixture theory framework to investigate hydraulic permeability and osmotically active intracellular water content. Hydraulic permeability and cell radius were significantly increased with LPS treatment and these changes were blocked in cells treated with TAK-242. LPS-induced remodelling of cortical actin and IL-6 upregulation were also mitigated by TAK-242 treatment. These findings indicated that TLR4 signalling participated in NP cell biophysical regulation and may be an important target for mitigating altered cell responses observed in IVD inflammation and degeneration.

## Introduction

Back pain stemming from IVD degeneration is a leading cause of disability worldwide and causes significant economic and societal burden (Cheung *et al.*, 2009; Diseases and Injuries, 2020; Hurwitz *et al.*, 2018). Back pain from DD is conventionally treated with immunosuppressive (steroid) injections or surgery, both of which are only partially effective (Lurie *et al.*, 2014; Weinstein *et al.*, 2007; Weinstein *et al.*, 2006; Weinstein *et al.*, 2008; Weinstein *et al.*, 2009; Weinstein *et al.*, 2010). Therefore, there is a significant clinical need for better approaches to mitigate degeneration and promote recovery of spine function. DD is defined by changes to ECM (Antoniou *et al.*, 1996; Keshari *et al.*, 2008; Millward-Sadler *et al.*, 2009; Pearce *et al.*, 1987; Roughley *et al.*, 2002), decreased disc height and altered mechanical function (Fujita *et al.*, 1997; Fujiwara *et al.*, 2000; Gu *et al.*, 1999a; Gu *et al.*, 1999b; Iatridis *et al.*, 2013; Johannessen and Elliott, 2005; O’Connell *et al.*, 2011). Proteoglycan degradation in DD occurs in part due to increased catabolic enzymes induced by inflammatory mediators (*e.g.* MMPs, ADAMTs). Increases in expression of NO, PGE2 (Kang *et al.*, 1996; Kang *et al.*, 1997; Liu *et al.*, 2001) and pro-inflammatory cytokines, especially TNFα, IL-1β and IL-6, have been shown to occur during DD (Ahn *et al.*, 2002; Bachmeier *et al.*, 2007; Le Maitre *et al.*, 2005; Le Maitre *et al.*, 2006; Le Maitre *et al.*, 2007; Seguin *et al.*, 2005; Shamji *et al.*, 2010; Zhang *et al.*, 2016). Recent studies have highlighted a significant role of innate immune activation, particularly of TLRs in DD (Klawitter *et al.*, 2014; Maidhof *et al.*, 2014; Quero *et al.*, 2013; Rajan *et al.*, 2013), where a pro-inflammatory signalling can trigger DD in the absence of a traumatic insult (Lai *et al.*, 2016; Rajan *et al.*, 2013). Activation of TLR4 by LPS has been shown to induce a pro-inflammatory cascade in IVD cells, with subsequent loss of IVD matrix integrity (Rajan *et al.*, 2013). TLRs are members of a receptor family activated by DAMPs that results in persistent pro-inflammatory signalling (Lin *et al.*, 2011; Risbud and Shapiro, 2014). While inflammation is a necessary step in wound healing, persistent inflammation can damage tissues and may contribute to pain (Tracey, 2007). IVD expression of TLR2 and TLR4 increases with increasing DD severity and mediates catabolic and inflammatory processes (Klawitter *et al.*, 2014; Maidhof *et al.*, 2014; Quero *et al.*, 2013; Rajan *et al.*, 2013).

Innate inflammation also has consequences on cell mechanobiology. Activation of TLR4 in NP cells from the gelatinous central region of the IVD alters the actin cytoskeleton and cell mechanotransduction by increasing cellular hydraulic permeability and cell size (Maidhof *et al.*, 2014). The cytoskeleton participates in sensing and transmission of mechanical signals from the ECM to the cells (Blain, 2009; Chen *et al.*, 2004; Hayes *et al.*, 1999; Li *et al.*, 2008). NP cells are spherical and their cytoskeleton is composed of actin microfilaments (F-actin), microtubules and vimentin intermediate filaments. NP cells are derived from the notochord and, in immature NP cells, F-actin has a punctuate structure that is constrained to the cell cortex (Li *et al.*, 2008). This diffuse cytoskeleton regulates the cell response to compressive loading (Chen *et al.*, 2004). NP cells are continuously subjected to hydrostatic and osmotic pressure induced by spinal loading, and mechanical loading in the disc is known to regulate cell volume (Mavrogonatou and Kletsas, 2012), gene expression (Neidlinger-Wilke *et al.*, 2006; Wuertz *et al.*, 2007), protein synthesis (Neidlinger-Wilke *et al.*, 2012; Sivan *et al.*, 2006) as well as being critical during growth and development (Urban, 2002). Maintenance of biomechanical properties at the cellular level also prevents degenerative changes within the disc at the tissue level (Hernandez *et al.*, 2020). Protection against changes in cellular mechanical properties, using inhibitors of actomyosin contractility, mitigates degenerative changes at the tissue level, including the prevention of GAG loss and inhibition of pro-inflammatory cytokine and MMPs expression (Hernandez *et al.*, 2020). As such, the maintenance of cellular mechanical properties of disc cells is a promising strategy for the prevention of degenerative effects within the disc.

The goal of the present study was to investigate the role of TLR4 in mediating cell morphological and cytoskeletal changes in NP cells and on biomechanical properties, specifically cellular hydraulic permeability. TLR4 activation has long-term consequences on NP cells, as cells do not spontaneously recover to baseline properties upon activation *in vitro* (Maidhof *et al.*, 2014). Therefore, the TLR4 inhibitor, TAK-242, was evaluated for protection against alterations of pro-inflammatory signalling, cell biomechanical properties and cytoskeleton. The study hypothesis was that TLR4 inhibition could protect against changes to NP cell biomechanical properties by mitigating alterations to the actin cytoskeleton and cell size.

## Materials and Methods

### Cell isolation and culture

NP tissue was harvested under sterile conditions from the lumbar spines (L1/2-L5/6) of 4 slaughtered juvenile cows (6–9 weeks old) that were obtained from an abattoir (Green Village Packing Company, Green Village, NJ, USA; permission was obtained to use these animal parts for research). Cells were isolated from pooled (L1/2-L5/6), minced NP tissue using 0.3 mg/mL collagenase type I + 0.3 mg/mL collagenase type II (Sigma) in complete medium [DMEM (Gibco) +10 % FBS (Atlanta Biologicals, Flowery Branch, GA, USA) + 1 % penicillin-streptomycin solution (Corning)] for up to 4 h. Then, digests were strained using a 70 μm cell strainer and cells were collected from digests by centrifugation (400 ×*g*, 5 min). Next, isolated cells were cultured in complete medium and standard culture conditions (37 °C, 5 % CO_2_) and expanded to passage 2 prior to further treatment and analysis. Medium was changed twice weekly.

### Part 1: evaluating dose-response of TAK-242 on LPS-stimulated NP cells

LPS (Sigma-Aldrich) was suspended in sterile dH_2_O by sonication (10 mg/mL) and diluted in DMEM to 0.1 μg/mL. TAK-242 (TLR4 inhibitor, EMD Millipore) was solubilised in pure DMF (Thermo Scientific) (25 mmol/L). TAK-242 stock was diluted in DMEM to create treatment solutions of varying doses: 0.1, 1, 10 or 100 μmol/L TAK-242. DMF equivalent to the highest concentration of TAK-242 (0.4 % DMF) was added to untreated controls and LPS groups to account for the possible effects of the solvent in TAK-242-treated groups.

To evaluate TAK-242 dose-response on LPS-stimulated cells, NP cells were transferred to 24-well plates (10^5^ cells/well) and cultured overnight in complete medium. Then, complete medium was replaced with either DMEM alone (untreated group), DMEM supplemented with LPS (0.1 μg/mL, LPS group) or DMEM supplemented with LPS (0.1 μg/mL) + TAK-242 at 0.1, 1, 10 or 100 μmol/L. In the LPS + TAK-242 groups, TAK-242 was added to the cells 1 h prior to the addition of LPS, to allow time for the intracellular inhibitor to act on the cells. Then, TAK-242 was maintained in the culture for 24 h along with the LPS co-treatment. After 24 h, medium supernatants were collected and assayed for nitrite and LDH release. Nitrite release, a breakdown product of released NO, was measured in cell supernatants by Griess reaction using a commercially available assay (Griess Reagent System, Promega), according to manufacturer’s protocol. The NO release response was analysed to determine the IC_50_, the concentration of TAK-242 inhibitor at which the NO release was reduced by half. The IC_50_ was determined as an absolute IC_50_ using a linear least square fit of percentage inhibition of NO release by TAK-242. Results were computed using GraphPad Prism software. LDH levels, an indicator of cell death, was measured using the Roche Cytotoxicity Detection Kit^PLUS^ according to manufacturer’s protocols. For LDH assay, a positive cytotoxic kill control was used by lysing an untreated group of cells with Triton X-100 (10×). Cytotoxicity (%) was determined using untreated and Triton X-100-treated cells as negative (0 %) and positive (100 %) cytotoxicity controls, respectively.

To further characterise the TAK-242 dose-response, *IL-6* expression was examined at the end of the 24 h treatment. Total RNA was extracted from treated NP cells using RNeasy kit (QIAGEN) following manufacturer’s instructions. Concentration and purity were measured by NanoDrop, with 260/280 ratios between 1.8 and 2.0 for all samples. Primers listed in Table 1 were designed using the Universal Probe Library v 2.45 (Roche). One-step quantitative PCR was performed using ABI7900 instrument and Eurogentec kit (RT-QPRT-032X, Eurogentec, Liège, Belgium) with 100 ng RNA and the following amplification protocol: 95 °C for 10 min followed by 40 cycles of 95 °C for 15 s and 60 °C for 1 min. Analysis of gene expression was performed with RQ Manager 1.2 software using the 2^− (ΔΔCt)^ method to calculate fold change relative to LPS treatment group.

To confirm the specificity of NP cell responses to LPS stimulation and not due to other potential contaminates in the culture, nitrite levels were measured in NP cells co-treated with PmB, an antibiotic that binds to LPS in solution. PmB (1, 25 and 50 μg/mL) was added to the medium 1 h prior to treatment of cells with LPS to ensure its binding to LPS.

### Part 2: evaluating the effects of TLR4 inhibition on cell mechanobiology

#### Biophysical properties and cytoskeletal imaging

**Fig. 1.**
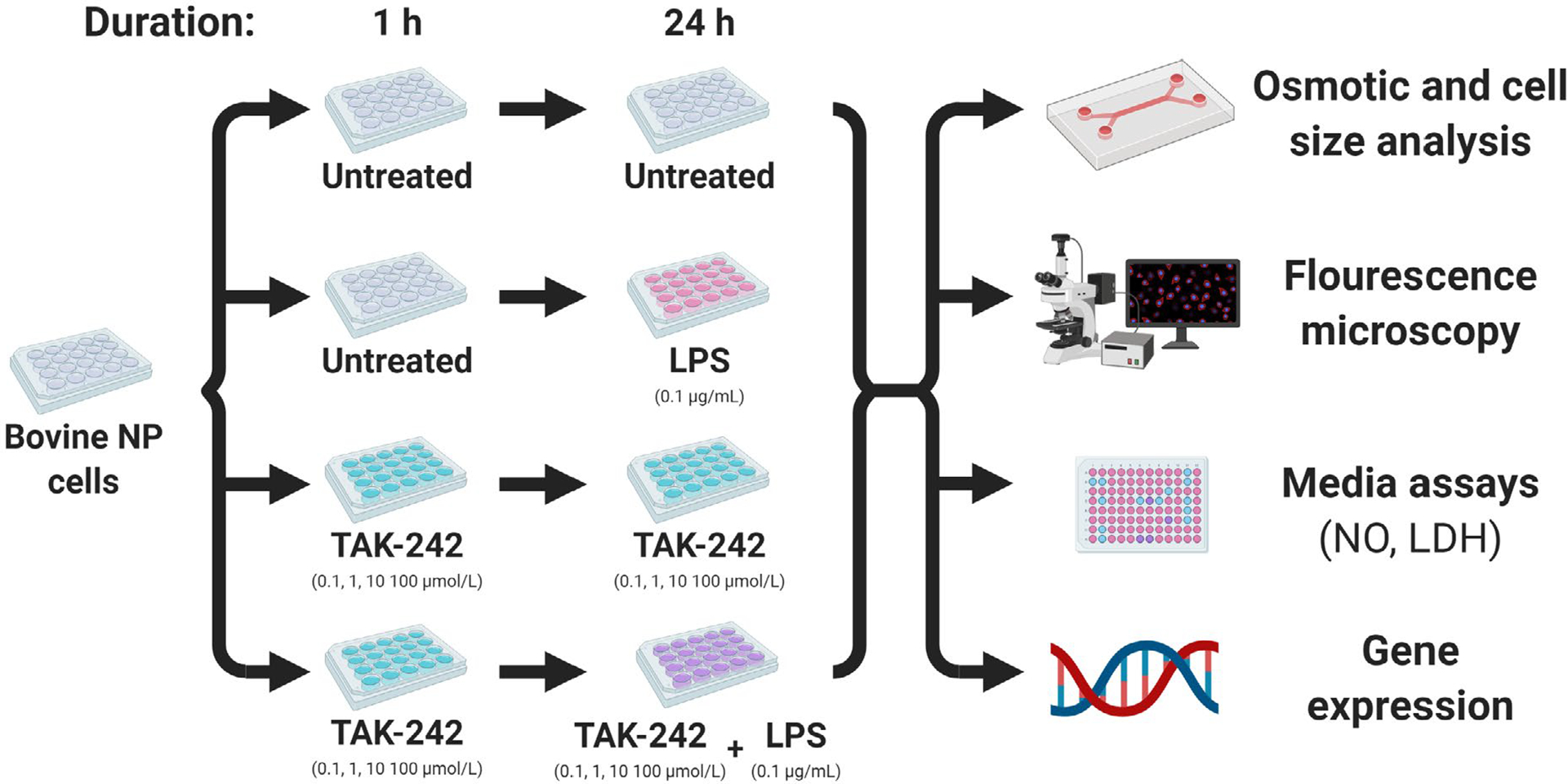
LPS and TAK-242 treatment study design and outcome measures. For part 2 studies, cells were tested under one of 4 treatment groups: (i) untreated, (ii) LPS, (iii) TAK-242, (iv) LPS + TAK-242. TAK-242 was introduced into the cell culture 1 h prior to 24 h co-treatment with LPS.

Based on part 1 results, subsequent experiments employed 1 μmol/L TAK-242 to achieve more than 50 % inhibition of TLR4 activation by LPS. In part 2 studies, NP cells were cultured in one of the following 4 groups: untreated, LPS (0.1 μg/mL), TAK-242 (1 μmol/L) or LPS + TAK-242 (Fig. 1). As in part 1, TAK-242 was added to the cells first as a pre-treatment, 1 h prior to the addition of LPS. Then, LPS or LPS + TAK-242 co-treatment was continued for 24 h.

NP cells from each treatment group were trypsinised and seeded in a custom-made Y-shaped microfluidic channel (Albro *et al.*, 2007; Chao *et al.*, 2005; Maidhof *et al.*, 2014). The PDMS channel (10 mm length, 300 μm width, 200 μm height) was sealed over a poly-D-lysine-treated glass slide. 40 μL of cell suspension were placed into the chamber. Then, the seeded cells were incubated at 37 °C for 30 min to allow attachment but retain a rounded morphology. Maintenance of cells in rounded morphology for this measurement was necessary to allow for accurate determination of cell volume by microscopy. Cells were equilibrated in a 333 mOsm/L NaCl solution for 5 min through the addition of 100 μL of 333 mOsm solution to each of the two upstream wells of the osmotic chamber. Loading was performed at room temperature to observe passive osmotic properties of the cells as cells do not exhibit active volume recovery when loaded in NaCl under these conditions and experimental duration (Albro *et al.*, 2009). After equilibration, a single hyper-osmotic loading step was applied (333 mOsm to 466 mOsm) followed by a hypo-osmotic loading step (466 mOsm to 333 mOsm) using NaCl solutions. These concentrations were selected based on typical osmolarities found within the disc *in vivo* (Urban and McMullin, 1988) and were used in prior studies (Hernandez *et al.*, 2020; Maidhof *et al.*, 2014). During osmotic loading, cells were imaged using an inverted confocal microscope using DIC filters. Images (0.2 μm/pixel) were acquired at a frequency of 0.5 Hz. A MatLab routine was used to segment individual cells and calculate a volume response over time for each cell (Maidhof *et al.*, 2014). The rounded morphology of imaged cells was used to compute cell volume from cross-sectional measurements. To track changes in basal cell radius due to treatments, NP cells post treatment were plated in the microfluidic chamber, incubated for 30 min and equilibrated at 333 mOsm NaCl. Due to the smaller effect size of treatment on radii measurements, a larger number of observations were made (*n* = 44–91 cells per group) when compared to the volume-response tests (*n* = 10–13 cells per group).

### Analysis of volume response

The cell volume response was analysed with the Kedem-Kachalsky model using a mixture theory framework (Albro *et al.*, 2007; Albro *et al.*, 2009; Ateshian *et al.*, 2006; Maidhof *et al.*, 2014). NaCl at room temperature is modelled as a non-permeating solute (Albro *et al.*, 2009; Bush and Hall, 2001), allowing the differential equation describing cell volume *V(t)* to be simplified to

$$\frac{dV}{dt}=AL_{p}R\theta \left(C_{i}-C_{e}\right)\;A=3\frac{V}{a}$$

where *A* is the volume-dependent cell surface area, *a* is the cell radius, *L*_*p*_ is hydraulic permeability, *R* is the universal gas constant, *θ* is the absolute temperature, *C*_*i*_ and *C*_*e*_ are the intracellular and extracellular osmolarities. *C*_*e*_ is defined by the experimentally applied osmotic solution, while *C*_*i*_ is governed by

$$C_{i}=\frac{n_{i}}{\Phi_{i}V}$$

where *n*_*i*_ is the number of moles of solute within the cell. Since NaCl is modelled as a non-permeating solute, *n*_*i*_ can be described by

$$\frac{dn_{i}}{dt}=0$$

where *Φ*_*i*_ is the volume fraction of osmotically active water in the cytoplasm that changes as water enters or leaves the cell according to the equation

$$\Phi_{i}=1-\left(1-\Phi_{ir}\right)\frac{V_{r}}{V}$$

The subscript *r* denotes the reference state of *V* and *Φ*_*i*_ (at the beginning of each loading step). Once the cell has reached equilibrium after an osmotic load is applied and

$$\frac{dV}{dt}\to 0$$

the reference intracellular water content (*Φ*_*ir*_) can be determined using the equation

$$\frac{V_{\infty }}{V_{r}}=1-\Phi_{ir}+\frac{\Phi_{ir}C_{er}}{C_{e}}$$

The measured volume response of each cell and osmotic step was curve-fit to this system of equations using a custom MatLab protocol to calculate values for *Φ*_*ir*_ and *L*_*p*_.

### Immunofluorescence

At end of treatment, some cells were trypsinised and plated on poly-L-lysine-coated glass coverslips for 25 min to maintain a rounded morphology. Cells were immediately fixed with 4 % paraformaldehyde in PBS for 10 min at room temperature, permeabilised with 0.1 % Triton-X100 in PBS for 10 min and blocked in 1 % BSA in PBS + 0.1 % Tween-20 for 1 h at room temperature. Primary antibodies anti-β-tubulin (1 : 1,000, Sigma, T8328) or anti-vimentin (1 : 1,000, Abcam, ab8069) were incubated in blocking buffer overnight at 4 °C. After three 5 min washes with PBS + 0.1 % Tween-20, samples were incubated with secondary antibody anti-mouse conjugated to Alexa 488 (1 : 500, A11029, Molecular Probes) and rhodamine-phalloidin (1 : 500, R415, Molecular Probes) in blocking buffer for 1 h at room temperature. Coverslips were again washed with PBS + 0.1 % Tween-20 for 5 min, mounted using Prolong Diamond (Thermo-Fisher Sci) and visualised in a Olympus XL70 laser scanning confocal microscope using a 40× objective.

### Gene expression

To further characterise the effects of TAK-242 in mitigating the response of LPS, the expression of pro-inflammatory, matrix degradation and ECM-related genes was examined at the end of the 24 h treatment period. Total RNA was extracted from treated NP cells using the QIAGEN RNeasy kit following manufacturer’s instructions. Concentration and purity were measured by NanoDrop, with 260/280 ratios between 1.8 and 2.0 for all samples. 200 ng of total RNA were converted to cDNA using the iScript cDNA Synthesis kit (BioRad). Primers listed in Table 2 were designed using the Integrated DNA Technologies PrimerQuest Tool (Integrated DNA Technologies). Quantitative PCR was performed using QuantiStudio 6 Flex system (Applied Biosystems, ThermoFisher Scientific) and iTaq Universal SYBR Green kit (BioRad) with the following amplification protocol: 95 °C for 30 s followed by 40 cycles of 95 °C for 15 s and 60 °C for 1 min. Analysis of gene expression was performed by QuantiStudio Real-Time PCR Software v1.3 software (Applied Biosystems) using the 2^− (ΔΔCt)^ method to calculate fold change relative to the untreated group.

### Statistical analysis

Results are presented as mean ± standard deviation, unless otherwise noted.

Part 1: NO release, LDH release and *IL-6* expression outcomes were analysed by one-way ANOVA, with TAK-242 or PmB dose as an independent variable. Pairwise comparison was made by Fisher LSD *post-hoc* test, with *p* < 0.05 considered statistically significant (Statistica, TIBCO Software, Palo Alto, CA, USA).

Part 2: osmotic analysis, cell size and gene expression data were analysed by two-way ANOVA for effect of LPS and TAK-242 as independent variables. Pairwise comparisons were made using the Fisher LSD *post-hoc* test, with *p* < 0.05 considered statistically significant (Statistica). Differences in hydraulic permeability and osmotically active water between hyper- and hypo-osmotic steps were tested for within each treatment group using unpaired *t*-test, with *p* < 0.05 considered statistically significant (Statistica).

## Results

### Part 1: evaluating dose-response of TAK-242 in LPS-stimulated NP cells

**Fig. 2.**
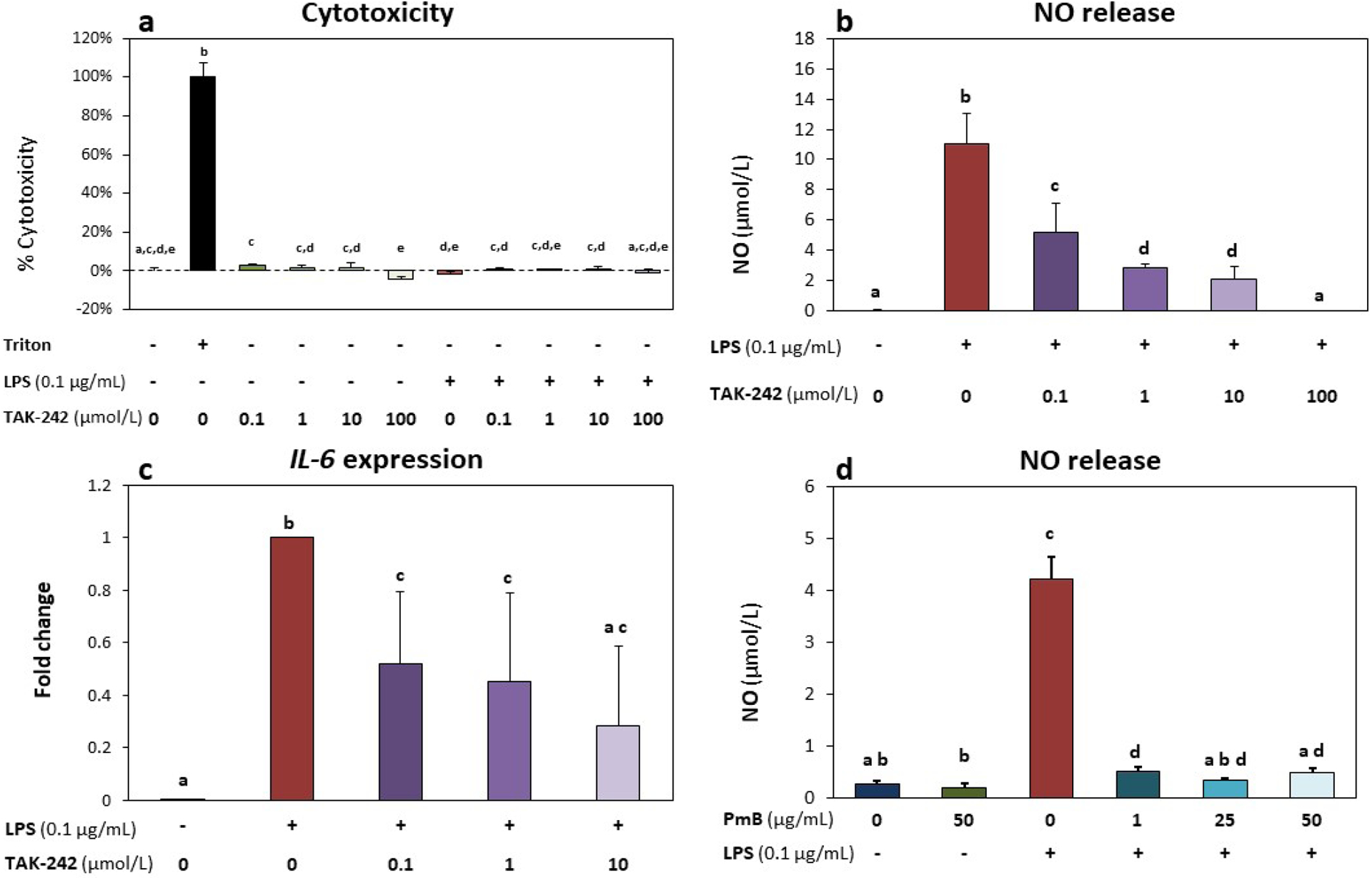
TLR4 inhibition mitigated LPS-induced inflammatory response. (**a**) Cytotoxicity (%) based on LDH levels did not increase with either LPS (0.1 μg/mL) or TAK-242 treatment, indicating no loss in cell viability in any of the experimental treatment group. Cells treated with 10× Triton X-100 for 10 min at the end of culture were used as a cytotoxic (kill/positive) control. (**b**) TAK-242 inhibited LPS-induced increases in NO release in a dose-dependent manner. (**c**) Cells treated with LPS showed significantly up-regulated expression of *IL-6*; TAK-242 co-treatment significantly decreased *IL-6* expression when compared to LPS-only group. Gene expression data shown as fold change *versus* LPS-only group. (**d**) NO release increased in cells treated with LPS. PMB inhibited LPS-induced increase in NO release. All groups were supplemented with 0.4 % DMF, equivalent to vehicle levels in the highest dose of TAK-242. All graphs show mean ± standard deviation (*n* = 3 biological replicates per group); groups with different letters indicate significant difference (*p* < 0.05) between groups.

No significant changes in cytotoxicity, as demonstrated by LDH levels, were observed in the LPS- or TAK-242-treated groups *versus* untreated control (*p* > 0.72) (Fig. 2a). Moreover, LDH levels in all groups were significantly lower than levels measured in the cytotoxic kill control group (*p* < 0.0001) (Fig. 2a). These results indicated that LPS with or without TAK-242 did not induce a significant loss of cell viability. Stimulation of cells with LPS significantly increased NO release (*p* < 0.05, Fig. 2b). Treatment of NP cells with the TLR4 inhibitor TAK-242 at 0.1, 1, 10 and 100 μmol/L inhibited LPS-induced NO release in a dose-dependent manner (*p* < 0.05, Fig. 2b). An IC_50_ of 0.0467 μmol/L TAK-242 was determined from a linear regression of normalised NO inhibition data (*R*^*2*^ = 0.80). TAK-242 dosages above the IC_50_ (0.1, 1, 10 and 100 μmol/L) all significantly reduced NO levels by 52.3 %, 74.3 %, 81.1 % and 101.5 %, respectively (Fig. 2b). Cells stimulated with LPS had significantly higher *IL-6* expression when compared to untreated cells (454 fold, *p* < 0.001). 0.1, 1 and 10 μmol/L TAK-242 had significantly lower *IL-6* expression *versus* LPS-only group (48.2 %, 55.0 % and 71.6 % reduction *vs.* LPS, respectively, *p* < 0.05, Fig. 2c). To confirm the specificity of LPS responses, NO release from cells co-treated with LPS and PmB was measured. Cells co-treatment with PmB + LPS at all tested doses had significantly lower NO release (1 μg/mL: 0.515 ± 0.082 μmol/L; 25 μg/mL: 0.332 ± 0.05 μmol/L; 50 μg/mL: 0.497 ± 0.082 μmol/L) compared to LPS-treated cells (4.212 ± 0.429 μmol/L, *p* < 0.05 for each dose group comparison). No significant difference in NO levels were observed between untreated cells (0.259 ± 0.077 μmol/L) and cells treated with LPS + 25 μg/mL PmB (*p* = 0.56) or LPS + 50 μg/mL PmB (*p* = 0.056). Cells treated with 50 μg/mL PmB alone also had comparable NO levels (0.204 ± 0.077 μmol/L) to untreated cells (*p* = 0.86, Fig. 2d), confirming that a potentially low (background) level of endotoxin was not a major factor in this culture model/system.

### Part 2: osmotic properties and equilibrium cell radius

**Fig. 3.**
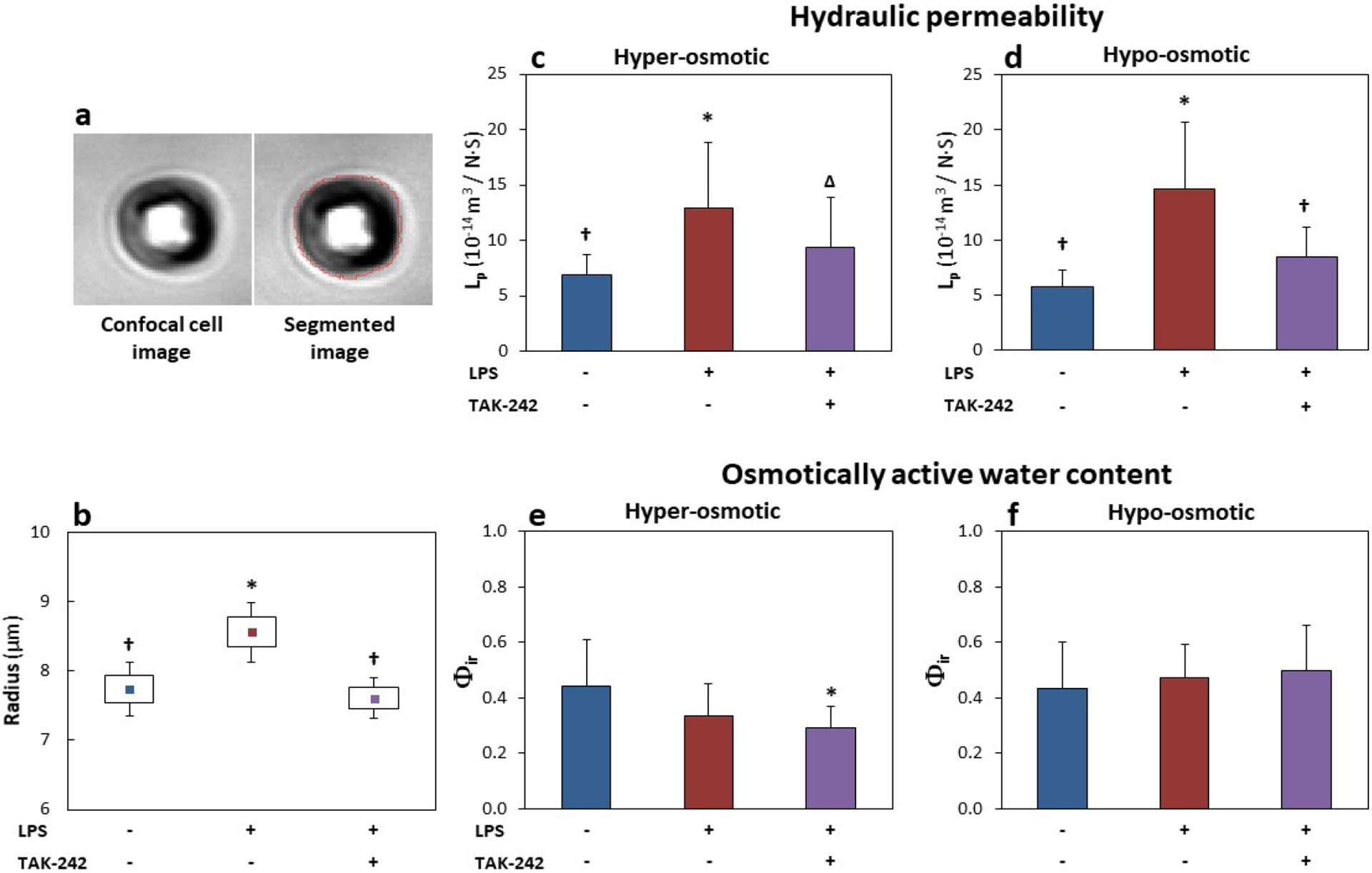
TLR4 inhibition mitigated LPS-induced changes in NP cell biophysical properties. (**a**) Representative DIC cell image used in cell radius and osmotic analysis and the resulting cell area attained by image segmentation (red line). (**b**) Cell radius measured at 333 mOsm/L increased with LPS treatment (0.1 μg/mL) and returned to untreated levels with addition of 1 μmol/L TAK-242. (**c**,**d**) L_p_ was significantly increased with LPS stimulation and returned to untreated levels with TAK-242 treatment for both (**b**) hyper- and (**c**) hypo-osmotic steps. (**e**,**f**) Φ_ir_ was only significantly affected in the LPS + TAK-242 co-treatment group for the hyper-osmotic step. (**d**) No other significant differences in Φ_ir_ were noted. All groups were supplemented with 0.4 % DMF. (**b**) Data show mean (▪), standard error (boxes) and 95 % confidence intervals (*n* = 44–91 cells per group). (**c**-**f**) Data show mean ± standard deviation (*n* = 8–13 cells per group). * *p* < 0.05 *vs.* untreated; ^†^ *p* < 0.05 *vs.* LPS alone; ^Δ^ *p* = 0.11 *vs.* LPS alone.

NP cell radius at isotonic osmolarity (333 mOsm/L NaCl) was found to increase with LPS (0.1 μg/mL) treatment. Significant increases in cell radius were found in cells treated with LPS (8.56 ± 1.52 μmol/L, *p* < 0.005) compared to untreated control (7.74 ± 1.59 μmol/L) (Fig. 3b). However, the radius of cells co-treated with TAK-242 + LPS (7.61 ± 1.41 μmol/L) was significantly lower than that of LPS-treated cells (*p* < 0.01) and showed no significant difference from that of untreated cells (*p* = 0.6).

L_p_ significantly increased with LPS stimulation in both hypo- and hyper-osmotic loading steps. Treatment with 1 μmol/L of TAK-242 significantly mitigated LPS-induced increase in L_p_ for the hypo-osmotic step. Co-treatment of cells with LPS + TAK-242 significantly decreased hypo-osmotic L_p_ compared to cells receiving LPS-only stimulation (Fig. 3c,d, Table 3). A trend for lower hyper osmotic L_p_ was observed in LPS + TAK-242 *vs.* LPS-only groups (*p* = 0.11), though this effect did not reach statistical significance. No significant differences in L_p_ were observed between untreated cells and TAK-242-treated groups. No significant differences in L_p_ were found between the hyper- and hypo-osmotic steps.

No significant changes in Φ_ir_ were found due to inflammatory stimulation with LPS (Fig. 3e,f; Table 4). Cells co-treated with LPS + TAK-242 had a small but significant reduction in Φ_ir_ in the hyper-osmotic step only.

### Part 2: cytoskeleton

**Fig. 4.**
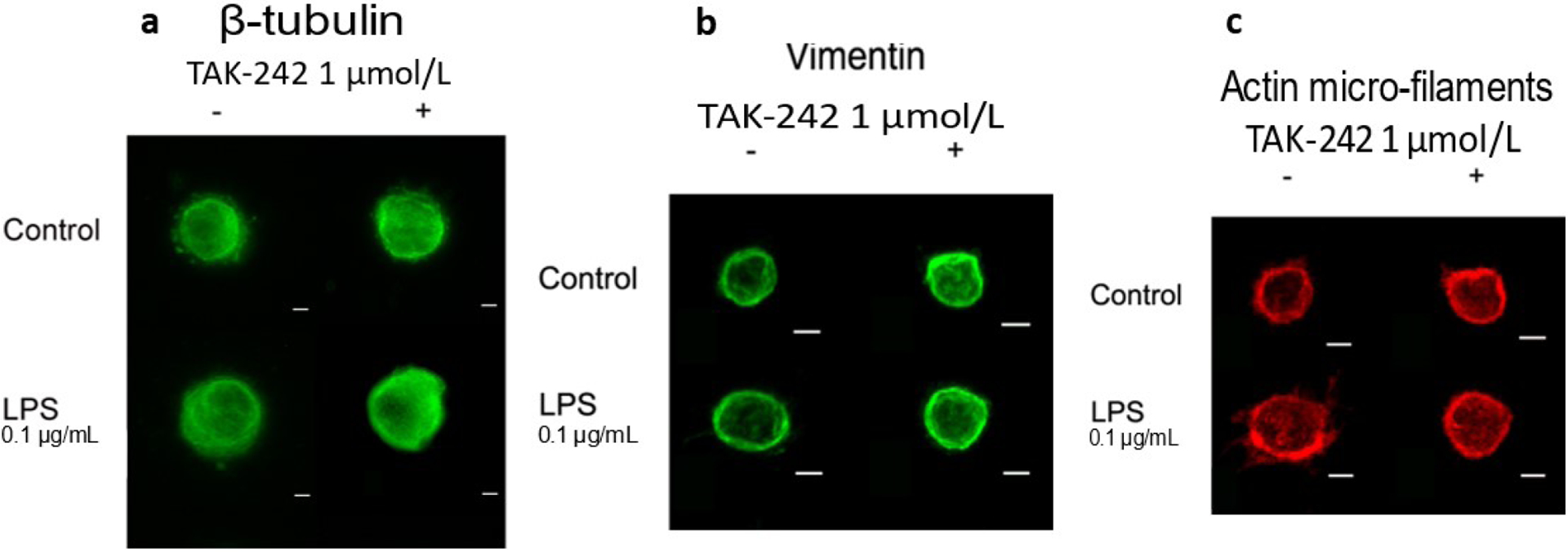
TLR4 inhibition mitigated LPS-induced cytoskeletal changes. (**a**) No changes in β-tubulin were observed with LPS or TAK-242 treatment. (**b**) No changes in intermediate vimentin filaments were observed with LPS or TAK-242 treatment. (**c**) Changes in F-actin (rhodamine-phalloidin) induced by LPS treatment were mitigated by TAK-242. Cells treated with LPS showed presence of small cell projections and increase in cortical actin fibres, which were prevented in cells co-treated with TAK-242. All groups were supplemented with 0.4 % DMF. Scale bars: 10 μmol/L; images are Z-projections.

Cytoskeletal changes were investigated in rounded cells from all groups. While no apparent changes to microtubules (Fig. 4a) and vimentin intermediate filaments (Fig. 4b) were observed, more evident changes were observed for actin microfilaments. In untreated NP cells, actin was distributed in a punctuate pattern, with no presence of stress fibres in the perinuclear area and few small cytoplasmic processes (Fig. 4c). NP cells treated with LPS exhibited increased cytoplasmic processes rich in F-actin, with no increase in actin fibres within the perinuclear area. The changes in F-actin due to LPS treatment were blocked by the addition of TAK-242. Cells pre-treated with TAK-242 showed no presence of projections and instead appear to have a more homogeneous distribution of F-actin at the cell cortex similar to that in untreated cells.

### Part 2: gene expression

**Fig. 5.**
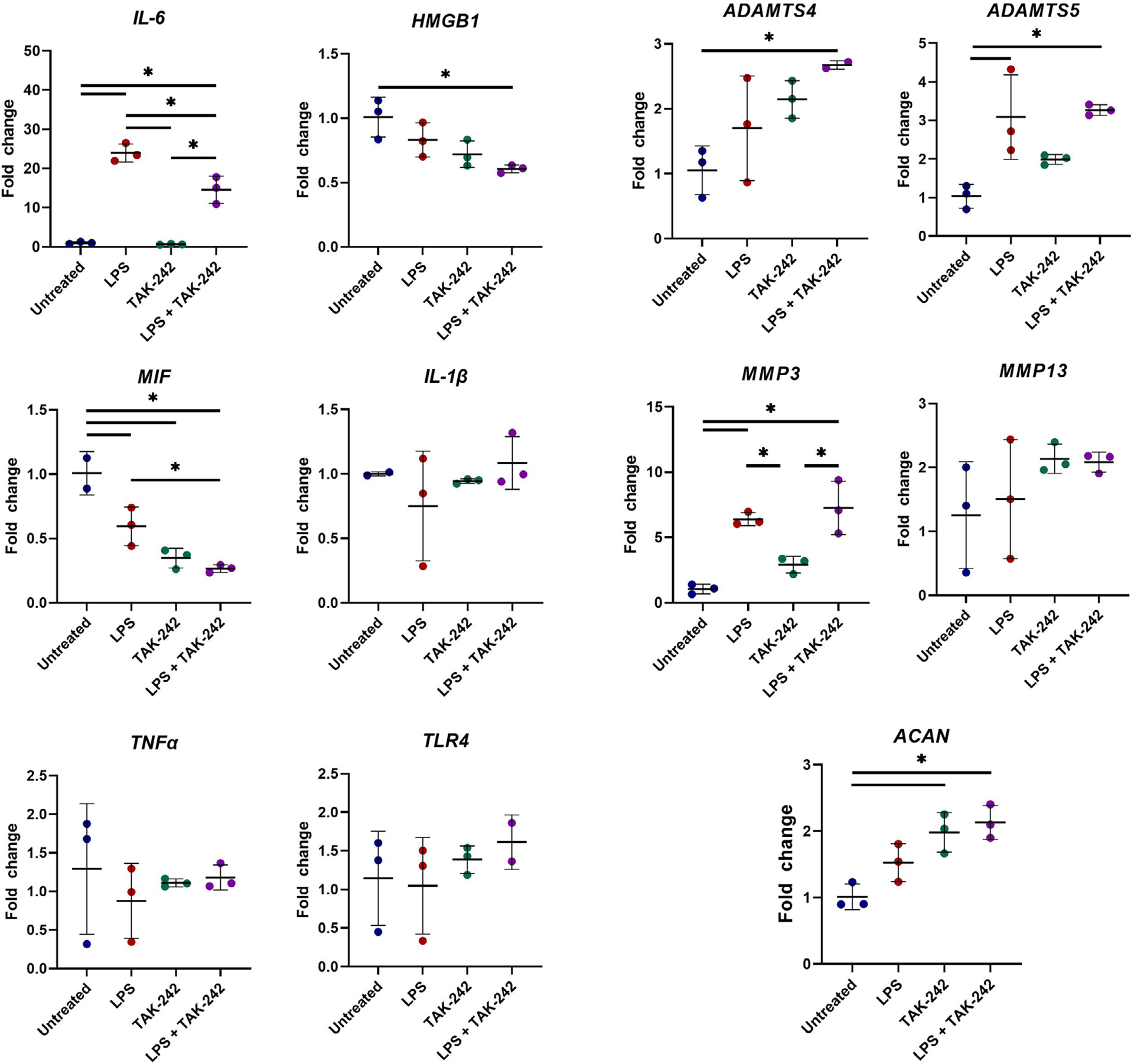
Gene expression of NP cells from part 2 study groups. All graphs showed gene expression fold change *versus* untreated. (Left) Gene expression of pro-inflammatory cytokines *IL-6*, *HMGB1*, *MIF*, *IL-1β*, *TNF-α*, and *TLR4*. (Right) Gene expression of matrix-degrading enzymes *MMP3*, *MMP13*, *ADATMS4*, *ADAMTS5* and ECM component *ACAN*. 0.1 μg/mL LPS significantly increased *IL-6*, *MMP3* and *ADAMTS5* expression. Addition of 1 μmol/L TAK-242 to LPS significantly reduced *IL-6* but not *MMP3* or *ADAMTS5* expression as compared to LPS alone. TAK-242 significantly increased *ACAN* expression as compared to untreated. No significant changes in *IL-1β*, *TLR4*, *TNFα* or *MMP13* were observed. All groups were supplemented with 0.4 % DMF. All graphs show mean ± standard deviation, with dots representing individual samples (*n* = 3 biological replicates per group). * *p* < 0.05 between indicated groups.

0.1 μg/mL LPS treatment was found to significantly increase *IL-6* expression, in agreement with gene expression from part 1 study (Fig. 2c). Co-treatment with LPS + TAK-242 significantly reduced *IL-6* expression by 40 % compared to LPS alone (Fig. 5). Both *HMGB1* and *MIF* expression was significantly reduced in LPS + TAK-242 co-treated cells *versus* untreated cells (*p* < 0.05 and *p* < 0.001, respectively). *MIF* expression also decreased with TAK-242 treatment alone *versus* untreated cells (*p* < 0.01). *MMP3* and *ADMATS5* significantly increased with LPS stimulation (*p* < 0.01 and *p* < 0.05, respectively); however, LPS + TAK-242 co-treatment did not significantly alter *MMP3* or *ADAMTS5* expression *versus* LPS alone (Fig. 5). *ACAN* expression was not affected by LPS stimulation but significantly increased with TAK-242 treatment (TAK-242 *vs.* untreated and LPS + TAK-242 *vs.* untreated, *p* < 0.01). *ADAMTS4* was significantly increased in LPS + TAK-242-treated cells *versus* untreated cells (*p* < 0.01) but no differences from untreated cells were observed in cells treated with LPS alone or TAK-242 alone. Lastly, no significant differences in *IL-1β*, *TLR4*, *TNFα* or *MMP13* were observed between any treatment group (Fig. 5).

## Discussion

The goal of the study was to investigate the role of TLR4 in mediating cell morphological, cytoskeletal and biomechanical changes in NP cells. The findings showed that stimulation of NP cells with LPS significantly increased hydraulic permeability and cell size and promoted formation of F-actin-rich cellular extensions. Blocking TLR4 signalling by TAK-242 mitigated LPS-induced changes in cell size and hydraulic permeability and protected against changes in actin cytoskeleton. Since *TLR4* expression was unchanged with stimulation or treatment, the changes observed with TAK-242 treatment were a consequence of altered TLR4 signalling rather than changes in expression level of the TLR4 receptor. NP cell hydraulic permeability correlates with cell size (Maidhof *et al.*, 2014), a relationship indicative of the cell’s ability to regulate cellular function in response to osmotic loading. Osmotic signals are important in the highly hydrated IVD, where reversible hydration changes that occur as part of the diurnal cycle of the spine or irreversible changes in hydration that occur with degeneration and GAG loss can alter NP cell gene expression (Wuertz *et al.*, 2007). The finding that TLR4 played a role in mediating changes in cell hydraulic permeability and actin cytoskeleton suggests that inflammatory stimulation has consequences on both IVD cell biology and cell biomechanics that are preventable by blocking intracellular TLR4 signalling. Indeed, TAK-242 significantly mitigated increases in both hydraulic permeability and cell size, further confirming their relationship in NP cells.

Actomyosin regulates cell volume changes in dividing cells and in response to osmotic pressure (Stewart *et al.*, 2011). Inflammatory treated cells had an increased cell size that was accompanied by a disrupted cortical actin cytoskeleton and extension of small cytoplasmic processes. Inhibiting cortical actomyosin contractility promotes the formation of cell processes rich in actin and increases cell hydraulic permeability (Hernandez *et al.*, 2020). This confirms that an intact actin cytoskeleton is required to produce the forces that maintain cell shape. Interestingly, aquaporin-1 expression does not increase in LPS- or TNFα-stimulated NP cells (Maidhof *et al.*, 2014). Conversely, LPS stimulation decreases aquaporin-1 protein expression. Decreased aquaporin protein expression may be a compensatory mechanism in response to increased hydraulic permeability.

In the present study, cytoskeletal evaluation was performed using the same cellular preparation (rounded NP cells) and state as used for the osmotic (permeability) measurement. The aim was to understand structure-function relationships between cytoskeletal changes and biomechanical properties in inflammatory conditions. Actin appeared to be the main cytoskeletal component affected by LPS stimulation, where no apparent changes in β-tubulin or vimentin were observed. This was mostly in agreement with a previous work showing no changes in β-tubulin, minor changes in vimentin and major changes in actin with TNFα inflammatory stimulation of bovine NP cells in a 3D environment (Hernandez *et al.*, 2020). These findings are not surprising as tubulin plays a major role in organelle distribution and cell division, while functions related to mechano-response are mostly performed by actin and vimentin intermediate filaments (Blain, 2009). Of these two, actin is more intimately connected to myosin and myosin contraction while vimentin acts as a more passive shape and load stabilizer. Hernandez *et al.* (2020) found dysregulation in the transcriptome of myosin II and Rho GTPase family members following inflammatory stimulation that ultimately decreased actomyosin’s contractility and subsequently altered cell biophysical properties. Given the role of actomyosin contractility in affecting cell biophysical properties, actin was the main cytoskeletal element relatively affected compared to tubulin or vimentin. While previous results indicated that vimentin is mildly affected by inflammatory stimulation (Hernandez *et al.*, 2020), in the context of LPS stimulation, no changes were observed.

Increasing actomyosin contractility is sufficient to prevent hydraulic permeability and actin cytoskeletal changes in TNFα-stimulated NP cells (Hernandez *et al.*, 2020). In the present study, both the increase in cell volume and actin alteration due to LPS stimulation could be blocked by TAK-242 treatment. This finding pointed to a relationship between TLR4 signalling and actin regulation in NP cells. Changes in NP cell morphology and cytoskeleton due to LPS treatment might results in lower compressive stiffness in NP cells, based on similar changes in hydraulic permeability, morphology and cytoskeleton as seen with TNFα stimulation (Hernandez *et al.*, 2020). Importantly, however, the effects of inflammation on cell biomechanics and cell cytoskeleton vary based on cell type and cell morphology. In monocyte and macrophages, studies indicate that TLR4 activation induces rapid and concentration-dependent increase in stiffness and increased F-actin staining (Doherty *et al.*, 1994; Pi *et al.*, 2014), while other studies find these cell types become considerably less stiff after stimulation (Leporatti *et al.*, 2006).

TAK-242 (resatorvid) is a selective intracellular inhibitor of TLR4 that does not inhibit an interaction between the ligand (LPS) and its receptor. TAK-242 binds to the intracellular TIR domain of TLR4 and blocks interaction with the adaptor proteins TRIP and TRAP (Ii *et al.*, 2006; Kawamoto *et al.*, 2008; Matsunaga *et al.*, 2011). TAK-242 inhibits production of inflammatory mediators including NO, IL-6 and TNFα in macrophages (Ii *et al.*, 2006; Matsunaga *et al.*, 2011; Sha *et al.*, 2007) and myotube cell lines (Hussey *et al.*, 2013; Ono *et al.*, 2020). TAK-242 treatment mitigates LPS-induced acute lung injury in mice (Seki *et al.*, 2010) as well as septic kidney injury in sheep (Fenhammar *et al.*, 2011). TAK-242 also inhibits expression of IL-6 and matrix-degrading enzymes including MMP-1, following stimulation with the DAMP HMGB1 in human NP cells (Shah *et al.*, 2019). Only a small number of clinical trials has evaluated TAK-242 in humans. Systemic TAK-242 administration was found to have a positive effect on traumatic brain injury recovery given systemic administration for 5 consecutive days after injury (Zhang *et al.*, 2014). However, another study investigating the use of TAK-242 failed to show TAK-242 efficacy for the treatment of sepsis (Rice *et al.*, 2010). LPS is recognised by TLR4 in a complex with MD2 and CD14 (Hoshino *et al.*, 1999; Nomura *et al.*, 2000; Poltorak *et al.*, 1998; Schumann *et al.*, 1990; Shimazu *et al.*, 1999; Wright *et al.*, 1990). The complex formed by TLR4-MD2-CD14 signals through a conserved pathway that results in the phosphorylation of NF-κB and initiates transcription of several pro-inflammatory cytokines such as IL-6 and TNFα (Goldring *et al.*, 1998; Kastenbauer and Ziegler-Heitbrock, 1999; Ziegler-Heitbrock *et al.*, 1994). These cytokines are responsible for the initiation of innate immune responses.

To characterise inflammatory response and signalling in the presence of LPS and TAK-242, gene expression of inflammatory factors (IL-6, IL-1β, TNFα, HMGB1, MIF), matrix degrading enzymes (MMP3, MMP13, ADMATS4, ADAMTS5) and ECM components (ACAN) was evaluated. *IL-6*, *MMP-3* and *ADAMTS5* expression significantly increased with LPS stimulation. However, only *IL-6* expression decreased with co-treatment with LPS + TAK-242 as compared to LPS only. Unlike the study by Rajan *et al.* (2013), no significant changes in *TNFα*, *IL-1β*, *HMGB1*, *ADAMTS4* or *MMP13* with LPS treatment were observed. This may be a consequence of the DMF solvent used in all groups in the present study that can exert an anti-inflammatory and anti-oxidant activity (Zhao and Wen, 2018). Decreased expression of *MIF* was seen with TAK-242 treatment when compared with untreated cells and in LPS + TAK-242 co-treated cells compared with LPS-treated cells, indicating that TLR4 inhibition decreased *MIF* expression. *ACAN* expression was also unaffected by LPS but increased by TAK-242 treatment, indicating inhibition of TLR4 may have a positive effect on ECM production. Results suggested IL-6 was the predominant pro-inflammatory factor affected in this setting. While TAK-242 had a protective effect against the pro-inflammatory mediators IL-6 and NO, it did not mitigate the LPS-induced upregulation of matrix-degrading enzymes in this setting. One limitation was that the 24 h timepoint studied may not be aligned with the temporal profile of certain pro-inflammatory cytokines or MMPs/ADAMTSs following LPS stimulation. Another limitation was that this data represented the gene expression of matrix degrading enzyme in NP cells cultured in a 2D environment absent of a native or *de novo* ECM, potentially confounding the interpretation on MMPs/ADAMTS gene expression, which also did not fully represent changes in enzyme level or activity.

A growing body of evidence suggests that TLR4 is involved in the pathogenesis of IVD (Gomez *et al.*, 2015; Klawitter *et al.*, 2014; Quero *et al.*, 2013). DAMPS such as HMGB1 or fibronectin fragments have degenerative effects in disc cells, including increased production of pro-inflammatory cytokine and matrix-degrading enzymes, mediated by signalling through TLRs, specifically TLR2 and TLR4 (Fang and Jiang, 2016; Krock *et al.*, 2017; Shah *et al.*, 2019). Furthermore, HMGB1 signalling has been shown to increase TLR2 and TLR4 expression (Fang and Jiang, 2016), suggesting a possible potentiation of signalling through TLRs. Lactoferrin can mitigate inflammatory disc signalling through TLR4 and, to a lesser extent, TLR2 (Kim *et al.*, 2013). Naturally derived substances such as curcumin and triptolide prevent inflammation-induced catabolic and anabolic changes through TLR inhibition (Klawitter *et al.*, 2012a; 2012b). Inhibition of MyD88, an adaptor of TLR2 and 4 signal transduction, also mitigates LPS-and IL-1β-induced upregulation of catabolic and inflammatory factors in the IVD (Ellman *et al.*, 2012). While these studies identify TLR4 to be a critical regulator of joint pathology, cell mechanobiology contributions to these degenerative changes are unknown. The present study confirmed that LPS-induced pro-inflammatory signalling altered cell mechanobiology and the actin cytoskeleton. Moreover, pharmacological interventions that mitigate pro-inflammatory signalling, and specifically inhibition of TLR4 signalling, can protect cell mechanobiological function and potentially improve the long-term homeostasis of cells in IVD tissues. TLR4 inhibition has also been shown to protect against inflammatory stimulation in human NP cells (Shah *et al.*, 2019), further enhancing the relevance of TLR4 inhibition *via* TAK-242 as a pharmacological intervention for human IVD degeneration. Interestingly, some studies have shown that stimulation of disc cells with ECM proteins, such as hyaluronic acid fragments and decorin, promotes pro-inflammatory signalling mediated by TLRs (Quero *et al.*, 2013; Zwambag *et al.*, 2020). However, whether TLR4 activation by ECM proteins induces cell biophysical changes is unknown. The preservation of cell biophysical properties has also been previously shown to protect against tissue-level degeneration and GAG loss (Hernandez *et al.*, 2020). The preservation of cell biophysical properties by TAK-242 demonstrates its translational potential for mitigating DD. Indeed, a study by Krock *et al.* (2018) supports this premise, where SPARC-null mice (a DD and low-back pain model) treated with systemic TAK-242 exhibited decreases in cytokine release and less pain-like behaviour.

Potential limitations of the study include the use of cell plating for expansion, which may alter NP cell phenotype (Horner *et al.*, 2002) as well as inflammatory response (Demoor-Fossard *et al.*, 1999). However, for osmotic and cytoskeletal analysis, NP cells were observed in a rounded morphology that more closely mimicked the 3D morphology of NP cells *in situ*. The rounded re-attached state was used in cytoskeletal evaluation to match the morphological, structure-function state that the cells were in during osmotic (permeability) measurement. The aim was to understand how cytoskeletal changes related to the biomechanical properties measured. Importantly cytoskeletal morphology was compared across all treatment groups in the same conditions, following the same trypsinisation process, and differences between groups represented differences due to treatment regardless of the effects of trypsinisation or reattachment. Changes in cell actin cytoskeletal morphology in trypsinised and rounded NP cells following treatment with both LPS and the inflammatory cytokine TNFα have been previously observed and reported on (Maidhof *et al.*, 2014), indicating that this method yields reproducible effects. Additionally, Hernandez *et al.* (2020) reported on the effect of inflammatory TNFα stimulation on cell actin cytoskeleton morphology in both trypsinised/rounded conditions as well as in adhered and spread 2D conditions, where similar disruptions to actin in both conditions were observed. Those findings are comparable to the ones presented in the present study. While detachment and re-attachment of NP cells and disruption of integrins following trypsinisation may affect the cell cytoskeleton, conclusions were based on treatment groups compared to one another where they all received the same cell processing. Therefore, the conclusions reached were sound and supported by the results of the study. Another limitation of the study was that osmotic properties of NP cells were observed in the absence of local pericellular and extracellular matrices, which may affect the ability of cells to swell, contributing to cell volume regulation (Cao *et al.*, 2011). Lastly, the effect of LPS on pro-inflammatory signalling was complicated by the effects of the DMF solvent, which can exert anti-inflammatory and anti-oxidant effects (Zhao and Wen, 2018).

## Conclusions

This study demonstrated that the TLR4 inhibitor TAK-242 could prevent LPS-induced changes to cellular biophysical properties of NP cells and block pro-inflammatory signalling of IL-6 and NO. Maintenance of cell biomechanical properties in the face of inflammatory insult mitigates tissue level DD (Hernandez *et al.*, 2020). Therefore, inhibition of the inflammatory cascade *via* TLR4 signalling that results in altered cell biophysical properties is a promising strategy for mitigating DD.

## Discussion with Reviewers

Reviewer: With regards to the TAK-242 concentration and exposure duration used in the study to achieve at least 50 % inhibition of TLR-4 activation by LPS and based on current evidence of the application of TAK-242 in humans, could TLR-4 inhibition with TAK-242 be a feasible treatment strategy for degenerative disc disease in the future?

Authors: To date, only a small number of clinical trials have evaluated TAK-242 effects in humans. Systemic TAK-242 administration was found to be tolerated in patients and efficacious for certain applications (Rice *et al.*, 2010; Zang *et al.*, 2014). Systemic administration of TAK-242 has also been found to decrease pain behaviour in a mouse model of DD (Krock *et al.*, 2018). However, the standing paradigm for DD treatment strategies, given the avascular nature of the IVD, is that drug delivery will require targeted approaches or localised intradiscal injections. Several targeted and extended-release drug delivery strategies, such as injectable hydrogel of microparticle drug carries, are being investigated for application to the IVD (Tryfonidou *et al.*, 2020, additional reference) and could allow for extended delivery of TAK-242. A recent study has shown that a nanostructure delivery system can promote solute retention within the IVD, which could allow for extended drug delivery for the treatment of DD (Wagner *et al.*, 2020, additional reference).

## Figures and Tables

**Table 1.** Part 1 primers.

Gene	Forward	Reverse
*IL-6*	TGATCCAGATCCTGAAGCAAA	CATCTTCTCCAGCAGGTCAGT
*GAPDH* (housekeeping)	GCATCGTGGAGGGACTTATG	GGGGCCATCCACAGTCTT

**Table 2.** Primers for gene expression.

Gene	Forward	Reverse
*IL-1β*	GTTAGAGTGCCATCCCTTCTGTC	CCATTGCCTTCTCCGCTATT
*IL-6*	CCAGAACGAGTATGAGGGAAATC	TTGTGGCTGGAGTGGTTATTAG
*TNFα*	GCCAACTCCCTCTGTTTATGT	GCCAACTCCCTCTGTTTATGT
*TLR4*	CATGGGCTTAGAGCAACTAGAA	GCGGAGGTTTCTGAGTGATAG
*HMGB1*	ACACTGCTGCGGATGATAAG	GGTTTCCCTTTAGCTCGGTATG
*MIF*	TGGCGCGCACAGATTC	GCGTCTCCACACCGTTTATT
*ADAMTS4*	AGACACAAGCAGGGAGAAAC	ACCTTCAGAGGAGTTGGAGA
*ADAMTS5*	GACCAGATTCACTGCCTACTT	CGTTGATGTCGATGATGGTTTC
*MMP3*	GTTGGTTTCTTCAGCACCTTTC	CAGCATCTCTGGGTAAATCCTT
*MMP13*	GAGCACTCATGTTTCCCATCTA	GAGTGGCAGTGGTGAATCTT
*ACAN*	GAGTGGCAGTGGTGAATCTT	CCACAGATCCTAAGCCTTCTTC
*GAPDH* (housekeeping)	GATGCTGGTGCTGAGTATGT	GCAGAAGGTGCAGAGATGAT

**Table 3.** L_P_ determined at each osmotic step.

L_p_ (10^−14^ m^3^/N·S)		
Treatment	Hyper-osmotic	Hypo-osmotic
Untreated	6.85 ±1.91	5.75 ± 1.54
LPS (0.1 μg/mL)	12.86 ± 5.93*	14.66 ± 6.08*
LPS + TAK-242 (1 μmol/L)	9.32 ± 4.56^Δ^	8.43 ± 2.72^†^

* *p* < 0.05 *vs.* untreated.

† *p* < 0.05 *vs.* LPS.

Δ *p* = 0.11 *vs.* LPS.

**Table 4.** Φ_ir_ determined at each osmotic step (mean ± standard deviation).

Φ_ir_		
Treatment	Hyper-osmotic	Hypo-osmotic
Untreated	0.441 ± 0.17	0.434 ± 0.17
LPS (0.1 μg/mL)	0.336 ± 0.12	0.472 ± 0.12
LPS + TAK-242 (1 μmol/L)	0.291 ± 0.08*	0.501 ± 0.16

* *p* < 0.05 *vs.* untreated.

## List of Abbreviations

ACAN: aggrecan
ADAMTS: a disintegrin and metalloproteinase with thrombospondin motifs
CD14: cluster of differentiation 14
DAMPs: damage-associated matrix proteins
DMEM: Dulbecco′s modified Eagle′s medium
DMF: dimethylformamide
DD: disc degeneration
DIC: differential interference contrast
ECM: extracellular matrix
FBS: foetal bovine serum
GAPDH: glyceraldehyde 3-phosphate dehydrogenase
HMGB1: high mobility group box protein 1
IC_50_: half maximal inhibitory concentration
IL: interleukin
IVD: intervertebral disc
LDH: lactate dehydrogenase
L_p_: hydraulic permeability
LPS: lipopolysaccharide
MD2: myeloid differentiation factor 2
MIF: macrophage migration inhibitory factor
MMPs: matrix metalloproteases
MYD88: innate immune signal transduction adaptor
NF-κB: nuclear factor kappa B
NO: nitric oxide
NP: nucleus pulposus
PDMS: polydimethylsiloxane
PGE2: prostaglandin E2
PmB: polymyxin B
SPARC: secreted protein acidic and rich in cysteine
TIR: toll/IL-1 receptor
TLR: toll-like receptor
TNFα: tumour necrosis factor alpha
TRAP: thrombospondin-related anonymous protein
TRIP: TNF-receptor-associated factor interacting protein
Φ: osmotically active water content
Φ_ir_: reference intracellular water content
